# COVID-19 Vaccine Hesitancy Among Pediatric Oncology and Bone Marrow Transplant Patients

**DOI:** 10.3390/vaccines12121407

**Published:** 2024-12-13

**Authors:** Sarah A. O’Neill, Son Tran, Cora Constantinescu, Tony H. Truong

**Affiliations:** 1 Department of Pediatrics, Alberta Children’s Hospital, University of Calgary, Calgary, AB T2N 1N4, Canada; sarah.oneill@ucalgary.ca; 2 Dalla Lana School of Public Health, University of Toronto, Toronto, ON M5S 1A1, Canada; 3 Section of Pediatric Infectious Diseases, Department of Pediatrics, Alberta Children’s Hospital, University of Calgary, Calgary, AB T2N 1N4, Canada; cora.constantinescu@albertahealthservices.ca; 4 Section of Pediatric Oncology and Cellular Therapy, Department of Pediatrics, Alberta Children’s Hospital, University of Calgary, Calgary, AB T2N 1N4, Canada

**Keywords:** vaccine hesitancy, vaccination, immunization, cancer, pediatric, immunocompromised, public health

## Abstract

**Background/Objectives:** Vaccine hesitancy among immunocompromised patients is complex and not well understood. This study aimed to determine the rate of COVID-19 vaccine hesitancy among pediatric oncology and bone marrow transplant (BMT) patients and to understand associated factors. **Methods**: Parents of patients (≤18 years) with cancer or post-BMT completed the Parent Attitudes about Childhood Vaccines Survey. A COVID-19 vaccine hesitancy score (VHS-COVID) was calculated from 0 to 100 (higher scores indicating increasing hesitancy). A small group of patients (patients older than 15 years) were also surveyed directly. **Results**: Among 113 parent respondents, the majority were female (58%) and at least college/university educated (78%). The majority (73%) of patients had cancer (61% leukemia/lymphoma, 37% solid/CNS tumors), while 27% had received BMT for malignant and non-malignant conditions. Only 48% of patients had been vaccinated against COVID-19, compared to 88% of parents. Ineligibility due to phase of cancer/BMT treatment (27%), vaccine hesitancy (24%), and age (24%) were the top three reasons for not vaccinating against COVID-19. Only 13% of parents said they would “definitely vaccinate” if their child became eligible. VHS-COVID scores were higher for parents of patients in surveillance versus active therapy (mean 61 vs. 48; *p* = 0.03). Parents who had received fewer COVID-19 vaccine doses (0–1 vs. ≥2) were more hesitant toward all vaccines (*p* = 0.0002), COVID-19 vaccines (*p* = 0.0003), and influenza vaccines (*p* = 0.005). **Conclusions:** Vaccine hesitancy is common among this population and was demonstrated through beliefs (hesitancy scores) as well as vaccine uptake. Future work should focus on education targeting vaccine eligibility and engaging with vaccine hesitant families in the immunocompromised community.

## 1. Introduction

The World Health Organization (WHO) has defined vaccine hesitancy as a ‘delay in acceptance of the vaccine despite the availability of vaccination services’ [1]. Addressing vaccine hesitancy becomes increasingly complex as we consider unique populations such as pediatric oncology and bone marrow transplant (BMT) patients, who have different vaccine-preventable disease risks and vaccine recommendations compared to the general population [2,3]. Vaccines are generally not provided to pediatric oncology patients during active chemotherapy or to BMT patients until 6 months post-transplant. Cytotoxic and myelosuppressive drugs limit the body’s ability to mount a robust immune response, thereby making routine vaccinations less effective in the immunocompromised host. Further, vaccination with live attenuated vaccines (e.g., measles, mumps, rubella, and varicella (MMRV) vaccine) are contraindicated due to the rare chance of developing an illness because of the vaccine [4].

In contrast, the pediatric and adult mRNA-based COVID-19 vaccines are not live vaccines, and although immunocompromised patients may not be able to mount a robust antibody response, possible anti-viral protection and desire to achieve greater herd immunity are desired benefits [5,6]. As such, COVID-19 vaccines are strongly recommended by national advisory committees on immunizations for immunocompromised patients such as cancer and bone marrow transplant patients [7].

Caregivers of childhood cancer survivors have expressed concerns about the COVID-19 vaccine due to the fragile health of their child, the risk of severe complications, inadequate safety information, and unclear necessity [8,9]. Parents have also reported greater hesitancy toward COVID-19 vaccines than routine childhood vaccinations, suggesting that using terms like “vaccine hesitant” may be more precise when referring to a specific vaccine [8].

We are beginning to understand COVID-19 vaccine hesitancy in survivor communities and the general public. However, the literature has yet to explore factors relating to vaccine hesitancy to COVID-19 among pediatric oncology patients undergoing active anti-cancer therapy, a time when they might be at increased severity risk from this disease [10,11,12]. Vaccine uptake rates have dropped across all childhood immunizations since the pandemic, with many sources citing a loss of confidence associated with the COVID-19 vaccine and COVID restrictions [13,14]. Understanding the hesitancy behind the COVID-19 vaccine may help guide us in understanding how to prevent the loss of confidence around other vaccines as well. For this study, we explored attitudes related to COVID-19 and influenza vaccines, with a brief inquiry into views around routine vaccinations in childhood. To compare parental views with patients, a small group of patients older than 15 years were also surveyed. By evaluating the factors affecting current (COVID-19) and annual respiratory seasonal (influenza) vaccinations, we can provide better information about perceived disease threat, vaccination effectiveness, and safety in our patients and develop strategies to increase vaccine uptake.

## 2. Materials and Methods

Pediatric oncology and bone marrow transplant patients and their parents were recruited from both the outpatient oncology/BMT clinic and the inpatient ward at the Alberta Children’s Hospital (ACH) in Calgary, Alberta, from February 2023 to September 2023. Parents were eligible to complete the survey if their child was under 18 years old, had a current or previous diagnosis of cancer, was in any stage of treatment/remission/follow-up, or had received a bone marrow transplant for any indication (malignant or non-malignant). Participants were excluded if they were unable to read/understand the English survey. Patients who had received the COVID-19 vaccine or had a previous confirmed diagnosis of COVID-19 were not excluded from the study. Patients older than 15 years were also allowed to complete the study to express their individual views. Informed consent was obtained at the start of the study questionnaire. The study was approved by the Health Research Ethics Board of Alberta: Cancer Committee.

Vaccine hesitancy was measured using an adapted version of the Parent Attitudes about Childhood Vaccines survey (PACV) by Opel et al. (2011) [15]. Survey questions maintained the same wording as the original instrument; however, participants were asked to answer the questions as they pertained to their views on a particular vaccine (i.e., routine childhood vaccinations, COVID-19, or influenza). Additionally, the word “shot” in the question stem was replaced by “vaccine”, as it is a more familiar term used in Canada. Survey responses were collapsed into three categories: hesitant, neutral/not sure, and non-hesitant. A vaccine hesitancy score was calculated by assigning a numeric score to each response: 2 points for each hesitant response, 1 for neutral/not sure, and 0 for each non-hesitant response. The responses of agree and strongly agree were combined together (Agree); disagree and strongly disagree were combined (Disagree). Hesitant reflects the combined responses of very and somewhat hesitant; not hesitant reflects the combined responses of not hesitant at all and not too hesitant. Concerned reflects the combined responses of very and somewhat concerned; not concerned reflects the combined responses of not concerned at all and not too concerned. For sliding scale questions (e.g., “From 0–10, how sure are you that following the recommended vaccine schedule is a good idea for your child?”), answers were collapsed into hesitant (0–3), not sure (4–7) and non-hesitant (8–10) categories. A raw total vaccine hesitancy score (VHS-Total) was calculated from the sum of each question score and transformed into a 0–100 scale, with higher scores indicating increased hesitancy. Separate scores were also tallied specifically for the COVID-19 and influenza vaccines and are referred to as “VHS-COVID” and “VHS-Flu”, respectively.

Potential associations with vaccine hesitancy scores (e.g., COVID vaccination status, treatment status, demographics) were assessed in bivariate analyses using *t*-tests, ANOVA, or Wilcoxon tests as appropriate to the data. Linear regressions were used to evaluate which demographic factors are associated with vaccine hesitancy scores. Predictors that met a significance threshold of *p* < 0.1 (two-tailed) were jointly evaluated in a multiple linear regression model. A manual stepwise backward elimination approach was used to identify significant covariates at a *p* < 0.05 (two-tailed) as required. Statistical analyses were conducted using the SAS statistical program (SAS-PC, version 9.4; SAS Institute Inc., Cary, NC, USA).

## 3. Results

### 3.1. Parent Respondents

Overall, 159 responses were received. Surveys were discarded due to respondents not consenting to the study (n = 4) or for not completing at least 2 sections of the survey (Routine Immunizations and Influenza; n = 22). Of the 133 remaining surveys, 113 were completed by parents. The remaining 20 surveys were completed by patients and analyzed separately. Two parents and one patient participant did not complete the final section on COVID-19, and statistics were adjusted accordingly. Among 113 parents, the majority were female (58%) and at least college/university educated (78%) (Table 1). Regarding their children, the majority (73%) were patients with cancer (61% leukemia/lymphoma, 37% solid/CNS tumors), while 27% had received BMT for malignant and non-malignant conditions. Most patients were undergoing active treatment (73%), with the remainder in surveillance/follow-up (27%).

Despite 56% of all parents reporting that they were at least somewhat hesitant about the COVID-19 vaccine, 88% of parents had received at least one dose (Table 2). In contrast, only 48% of their children had received at least one dose of the COVID-19 vaccine. Ineligibility due to stage of cancer/BMT treatment (27%), vaccine hesitancy (24%), and age (24%) were the top three reasons for not vaccinating their children against COVID-19. Only 13% of parents said they would “definitely vaccinate” their child if they became eligible. Overall, one-quarter of parents were unsure if they can trust the information they receive about COVID-19 vaccines, but the majority (66%) felt they could openly discuss these concerns with their child’s doctor.

Hesitant views were unique for some participant subgroups and, at times, only expressed toward certain vaccines. Families in the surveillance/follow-up subgroup were more hesitant toward the COVID-19 vaccine compared to those still in the in active treatment subgroup (VHS-COVID, 61 vs. 48; *p* = 0.03), but this difference was not significant for VHS-Total (*p* = 0.1) or VHS-Flu (*p* = 0.13; Table 3). Lower household income (<$50,000) was associated with increased hesitancy toward routine immunizations (*p* = 0.04) as well as flu vaccines (*p* = 0.04), but not for hesitancy toward the COVID-19 vaccine (*p* = 0.57).

Parents who were not regular recipients of the annual influenza vaccine (i.e., received two or fewer in the last 5 years) were generally more hesitant (though not significantly) toward all vaccine categories (VHS-Total, *p* = 0.14; VHS-COVID, *p* = 0.15; and Influenza-VHS, *p* = 0.09), compared to those who received three or more annual influenza vaccines. The following variables were not significantly associated with vaccine hesitancy (VHS-COVID, VHS-Flu, or VHS-Total) for parent participants: treatment type (cancer vs. BMT), cancer type, relapse status, or additional parental demographic factors (gender, parental education, child’s school).

In the multivariable linear regression, only COVID-19 vaccination status was a significant predictor of VHS-Total scores (parents who had one or fewer doses were more hesitant than those with two or more doses, beta 17.44, *p* = 0.0086). For VHS-COVID scores, parents whose children were in surveillance/follow-up were more hesitant compared to those in active therapy (beta 16.24, *p* = 0.047), and those having received one or fewer doses of COVID-19 vaccine (beta 19.33, *p* = 0.01). For the VHS-Flu scores, those with lower household incomes were more hesitant than those with higher incomes (beta 15.88, *p* = 0.04).

### 3.2. Patient Respondents (Older than 15 Years)

Patients older than 15 years had an opportunity to respond to the survey on their own (n = 20). Patient respondents were more balanced in gender split (53% female, 42% male) compared to parent respondents. Similar to the parent population, the majority of respondents were diagnosed with cancer (80%), though over half of the patient group (55%) were in surveillance/follow-up rather than active treatment (45%) (Table 1). Patients self-reported better COVID-19 vaccine uptake (85% had ≥2 doses) compared to parent respondents (69% of parents and 34% of their children had ≥2 doses). This was also reflected in patients having significantly lower vaccine hesitancy scores than parents for VHS-Total (29 vs. 47; *p* = 0.0036) and VHS-COVID (24 vs. 51; *p* = 0.0005) (Table 4).

Personal uptake of the influenza vaccine was similar between parent and patient respondents (50% were vaccinated in ≥3 of the last 5 years); however, their views about the vaccine differed. Parents were more hesitant than patients about the influenza vaccine (44% vs. 20% at least somewhat hesitant, respectively), despite both groups agreeing that the vaccine prevents serious illness. Further, many parents agreed it was better for their children to gain immunity by being sick with influenza rather than getting the vaccine (48% vs. 15% of patients). Overall, parents were more hesitant toward the influenza vaccine (VHS-Flu, 46 vs. 34 for patients); however, this was not significant (*p* = 0.09).

Among the patient respondents, the type of treatment received and COVID-19 vaccination status significantly affected hesitancy toward all vaccine categories (Table 5). Patients who received ≥2 doses of the COVID vaccine were significantly less hesitant toward all vaccine types (*p* = 0.02), COVID-19 vaccines (*p* = 0.02), and influenza vaccines (*p* = 0.03) than those who were unvaccinated against COVID-19 or had only received one dose. Individuals treated for cancer (n = 16) were less hesitant than patients who received BMT (n = 4); however, these data should be interpreted with caution given small numbers (*p* = 0.005, *p* = 0.02, *p* = 0.003 for VHS-Total, VHS-COVID, and VHS-Flu, respectively). The following variables were not significantly associated with vaccine hesitancy (VHS-COVID, VHS-Flu, or VHS-Total) for patient respondents: treatment phase, cancer type, relapse status, influenza vaccine status, or additional demographic factors (gender, parental education, school, household income).

## 4. Discussion

Parents of children with cancer and recipients of BMT exhibit hesitancy toward the COVID-19 vaccine despite the best evidence and guidelines supporting their use in this population. The strongest predictor of vaccine hesitancy was the number of COVID-19 vaccine doses received by the parent themselves. Overall, COVID-19 vaccination status, household income, and phase of cancer/BMT treatment were significantly associated with hesitancy scores in Total-VHS, VHS-COVID, and VHS-Flu. Patient respondents displayed less hesitancy than parent respondents for VHS and VHS-COVID.

This study builds on previous work in the survivorship community by including patients undergoing active treatment and was conducted three years into the pandemic to gain insight into belief systems as they have evolved over time. Supporting the work of Wimberley et al., parents surveyed had received more vaccines than their children [9]. Specifically, Wimberly et al. found that 29% of parents were hesitant to vaccinate their childhood cancer survivors against COVID-19, including 11% who would “definitely not” vaccinate. In contrast, only 20% of caregivers were hesitant to vaccinate themselves, and 12% of caregivers would vaccinate their other children differently than the childhood cancer survivor [9]. Contradictory to this, however, our study reports that patients themselves expressed less vaccine hesitancy than their parents toward all vaccine categories. This presents an interesting opportunity for targeting vaccine education toward patients aged 15 years and older, as they begin to express interest in their healthcare decisions and as in some places, such as Alberta, can make autonomous decisions regarding this.

Several studies in adult oncology have demonstrated the prevalence of COVID-19 vaccine hesitancy, up to 42% in a large French survey [16,17,18]. One study on adolescent and young adult cancer survivors reported that 24% of patients were hesitant about the COVID-19 vaccine, citing lower perceived disease vulnerability and concerns about vaccine safety [19].

Vaccines differ in their history (i.e., well-established, novel, or experimental), how they are perceived (i.e., effectiveness, necessity, and severity of side effects), and who they are offered to (i.e., population-wide or targeted toward specific subgroups). In this study, COVID-19 vaccination status was the strongest predictor of parental vaccine hesitancy (routine and COVID-19 vaccines), suggesting that beliefs and actions around COVID-19 vaccines are closely correlated. However, the same was not true for influenza vaccination status and hesitancy toward influenza vaccines. In addition to concerns about the vaccine itself, the threat of disease perceived by an individual is another important factor to consider. In our study, most parents agreed that influenza posed a serious disease threat (56%); however, many (48%) felt their child would be better off getting sick with the flu than vaccinating against it. There is a higher prevalence of the latter parental belief in our current study (48%) than what was observed from the knowledge, attitudes, and beliefs (KAB) study from the 2017 childhood National Immunization Coverage Survey (cNICS) in Canada. The cNICS, which surveyed Canadian parents about general childhood vaccines, reported that 31% of parents agreed that it is better for children to develop their immunity from natural infections rather than from vaccines [20]. Health care providers should recognize that complacency can often play a role in vaccination status and highlight the risk of disease in their discussions with families.

Parents of children in surveillance were more hesitant toward the COVID-19 vaccine than those in active treatment, but the same was not true toward the influenza vaccine. This supports the work of Temsah et al., who reported that individuals may express increased hesitancy toward some vaccinations and not others [8]. As such, vaccine hesitancy cannot be a blanket term applied to an individual’s views on all vaccinations, but factors related to hesitancy and complacency must be explored as they relate to each specific vaccine.

This study was limited by recruiting participants from only one center in Canada. This study took place at the Alberta Children’s Hospital, which serves a diverse patient population and is the only pediatric bone marrow transplant center in the province (and one of six in Canada). The survey was only available in English; thus, the data do not include the views of non-English speaking families. Due to the small sample size in our study and lack of statistical power, we may not have been able to detect true differences in both the parent and patient respondents. Additional patient recruitment would allow for a more robust comparison of vaccine hesitancy among patients and parents. The strengths of this study include the opportunity to explore COVID-19 vaccine hesitancy and compare it with routine childhood vaccines and the annual influenza vaccine. We were also able to survey a small group of patient respondents who had unique views regarding vaccine hesitancy compared to parent respondents.

Finally, this study has provided insight into opportunities for specific education with families around vaccine eligibility. The top three reasons parents selected for not vaccinating their child against COVID-19 were vaccine hesitancy and ineligibility due to age or stage of treatment. The COVID-19 vaccine is widely recommended to all immunocompromised patients, and only Canadians less than 6 months old are ineligible for the vaccine due to age. Most parents felt comfortable discussing vaccines with their doctor, which provides an excellent opportunity to address this type of vaccine misinformation and discuss hesitancy.

COVID-19 vaccines were introduced to Canadian adults in December 2020, shortly after the pandemic was declared in March 2020. For pediatric patients, it was not until November 2021 that children 5 years and older became eligible for mRNA vaccines. Lastly, children aged 6 months to 5 years old became eligible in July 2022 [21]. Despite two years of access to the vaccine, a large discrepancy between vaccinated children and adults remains. As of June 2024, only 8% of children aged 0–4 have received at least one dose of the vaccine, compared to 81% of all Canadians [22]. Patients under 6 months old remain ineligible for vaccination.

## 5. Conclusions

The pediatric oncology and BMT populations are vulnerable to diseases not only in the event of a future pandemic but also rely on immunity against routine diseases. Focusing on the risk of illness and the importance of disease prevention is paramount in such a vulnerable patient population. Since vaccine hesitancy varies depending on the disease and vaccine type, factors related to hesitancy should be openly explored both in general and for each vaccine type.

## Figures and Tables

**Table 1:** Participant demographics and details about the patient’s treatment.

	Number (%) of Parent Participants (n = 113)	Number (%) of Patient Participants (n = 20)
Respondent Gender		
Male	43 (39)	8 (42)
Female	64 (58)	10 (53)
Prefer not to disclose	3 (3)	1 (5)
Parental Education		
Master’s, Ph.D., professional degree	11 (10)	4 (21)
University degree	33 (30)	2 (11)
College diploma	42 (38)	3 (16)
High school	17 (15)	5 (26)
Below high school	2 (2)	5 (26)
Prefer not to disclose	5 (5)	
Type of Treatment Received		
Cancer	82 (73)	16 (80)
BMT (total)	31 (27) (18 malignant; 13 non-malignant)	4 (20) (all 4 non-malignant)
Treatment Status		
Active	83 (73)	9 (45)
Surveillance	30 (27)	11 (55)
Cancer Type		
Leukemia	36 (36)	4 (25)
Lymphoma	25 (25)	4 (25)
Brain tumor	9 (9)	1 (6)
Solid tumor	28 (28)	7 (44)
Other	2 (2)	0
Relapse status		
Yes	47 (47)	3 (18)
No	49 (49)	14 (82)
Unsure	4 (4)	
Patient’s School		
Private school	13 (12)	2 (11)
Public school	78 (71)	16 (84)
Homeschool	4 (4)	1 (5)
Not school age	10 (9)	n/a
Other	5 (5)	
Household Income		
>$100,000	35 (32)	11 (58)
$50,000–100,000	20 (18)	3 (16)
<$50,000	45 (41)	2 (11)
Prefer not to disclose	10 (9)	3 (16)
Patient COVID Vaccine status		
0 doses	59 (52)	3 (15)
1 dose	16 (14)	0 (0)
2 doses	29 (26)	7 (35)
3 or more doses	9 (8)	10 (50)
Parent COVID Vaccine status		
0 doses	14 (12)	n/a
1 dose	19 (17)	n/a
2 doses	48 (42)	n/a
3 or more doses	31 (27)	n/a
Unsure	1 (1)	n/a
Influenza Vaccine status	Parent	Patient
3–5 times in the last 5 years	57 (50)	10 (50)
Never/0–2 times in the last 5 years	56 (49)	10 (50)

Percentages may not total 100% due to rounding.

**Table 2:** Modified PACV survey questions and parent and patient responses.

Question	Response	Parents (n = 113) N (%)	Patients (n = 20) N (%)
Part 1: Views on Vaccines in General			
1. If you had another infant today, would you want him/her to get all the recommended vaccines?	Yes	81 (72)	n/a
	Not sure	23 (20)	n/a
	**No**	**9 (8)**	**n/a**
2. Children get more vaccines than are good for them	Disagree	40 (35)	17 (85)
	Not sure	19 (17)	2 (10)
	**Agree**	**54 (48)**	**1 (5)**
3. It is better for my child to develop immunity by getting sick than to get a vaccine	Disagree	40 (35)	15 (75)
	Not sure	19 (17)	0
	**Agree**	**54 (48)**	**5 (25)**
4. Overall, how hesitant about childhood vaccines would you consider yourself to be?	**Hesitant**	**37 (33)**	**4 (15)**
	Not sure	11 (10)	1 (5)
	Not hesitant	65 (58)	15 (75)
5. How sure are you that following the recommended vaccine schedule is a good idea for your child?	**0–3 (Not sure)**	**14 (12)**	**2 (10)**
	4–7 (Neutral)	50 (44)	8 (40)
	8–10 (Extremely sure)	49 (43)	10 (50)
Part 2: Views on Annual Influenza Vaccine			
1. Have you ever delayed your child getting *the annual influenza* vaccine for reasons other than illness, allergy, or your child’s cancer diagnosis?	**Yes**	**58 (51)**	**10 (50)**
	Not sure	5 (4)	2 (10)
	No	50 (44)	8 (40)
2. Have you ever decided not to get *the annual influenza* vaccine for your child for reasons other than illness, allergy, or your child’s cancer diagnosis?	**Yes**	**64 (57)**	**12 (60)**
	Not sure	4 (4)	2 (10)
	No	45 (40)	6 (30)
3. Overall, how hesitant about the influenza vaccine would you consider yourself to be?	**Hesitant**	**50 (44)**	**4 (20)**
	Not sure	13 (12)	3 (15)
	Not hesitant	50 (44)	13 (65)
4. I believe that the illness that the influenza vaccine prevents is severe	**Disagree**	**28 (25)**	**6 (30)**
	Not sure	22 (19)	4 (20)
	Agree	63 (56)	10 (50)
5. It is better for my child to develop immunity by getting sick with influenza than to get a vaccine	Disagree	39 (35)	14 (70)
	Not sure	20 (18)	3 (15)
	**Agree**	**54 (48)**	**3 (15)**
6. I trust the information I receive about the influenza vaccines	**Disagree**	**17 (15)**	**5 (25)**
	Not sure	36 (32)	2 (10)
	Agree	60 (53)	13 (65)
7. I am able to openly discuss my concerns about influenza vaccines with my child’s doctor	**Disagree**	**14 (12)**	**1 (5)**
	Not sure	22 (19)	3 (15)
	Agree	77 (68)	16 (80)
8. How concerned are you that your child might have a serious side effect from the influenza vaccine?	**Concerned**	**56 (50)**	**6 (30)**
	Not sure	14 (12)	4 (20)
	Not concerned	43 (38)	10 (50)
9. How concerned are you that the influenza vaccine might not prevent the disease?	**Concerned**	**55 (49)**	**4 (20)**
	Not sure	21 (19)	3 (15)
	Not concerned	37 (33)	13 (65)
Part 3: Views on COVID-19 mRNA vaccine			
1. Have you ever delayed your child getting the *COVID-19* vaccine for reasons other than illness, allergy, or your child’s cancer diagnosis?	**Yes**	**61 (55)**	**7 (37)**
	Not sure	3 (3)	2 (11)
	No	47 (42)	10 (53)
2. Have you ever decided not to get the *COVID-19* vaccine for your child for reasons other than illness, allergy, or your child’s cancer diagnosis?	**Yes**	**59 (53)**	**3 (16)**
	Not sure	3 (3)	1 (5)
	No	49 (44)	15 (79)
3. Overall, how hesitant about the COVID vaccine would you consider yourself to be?	**Hesitant**	**62 (56)**	**4 (21)**
	Not sure	9 (8)	0
	Not hesitant	40 (36)	15 (79)
4. I believe that the illness that the COVID vaccine prevents is severe	**Disagree**	**29 (26)**	**2 (11)**
	Not sure	18 (16)	2 (11)
	Agree	64 (58)	15 (79)
5. It is better for my child to develop immunity by getting sick than to get a COVID vaccine	Disagree	40 (36)	16 (84)
	Not sure	21 (19)	1 (5)
	**Agree**	**50 (45)**	**2 (10)**
6. I trust the information I receive about COVID vaccines	**Disagree**	**31 (28)**	**3 (16)**
	Not sure	28 (25)	2 (11)
	Agree	52 (47)	14 (74)
7. I am able to openly discuss my concerns about the COVID vaccine with my child’s doctor	**Disagree**	**21 (19)**	**1 (5)**
	Not sure	17 (15)	5 (26)
	Agree	73 (66)	13 (68)
8. How concerned are you that your child might have a serious side effect from a COVID vaccine?	**Concerned**	**72 (65)**	**5 (26)**
	Not sure	11 (10)	2 (11)
	Not concerned	28 (25)	12 (63)
9. How concerned are you that a COVID vaccine might not prevent the disease?	**Concerned**	**67 (60)**	**8 (42)**
	Not sure	13 (12)	0
	Not concerned	31 (28)	11 (58)

Hesitant responses are bolded. Survey questions for patients were modified to be directed to them instead of their child. Agree reflects the combined responses of strongly agree and agree; disagree reflects the combined responses of strongly disagree and disagree. Hesitant reflects the combined responses of very and somewhat hesitant; not hesitant reflects the combined responses of not hesitant at all and not too hesitant. Response category on a 0–10 scale, with 0 being ‘not at all sure’ and 10 being ‘extremely sure’. Concerned reflects the combined responses of very and somewhat concerned; not concerned reflects the combined responses of not concerned at all and not too concerned. Percentages may not total 100% due to rounding.

**Table 3:** Hesitancy outcomes for parent respondents based on demographics.

	VHS-Total		VHS-COVID		VHS-Influenza	
	Score, Mean (Median)	*p*-Value	Score, Mean (Median)	*p*-Value	Score, Mean (Median)	*p*-Value
Parental Gender						
Male	48 (54)	0.99	50 (61)	0.61	48 (56)	0.63
Female	47 (52)		53 (64)		45 (50)	
Parental Education						
High	47 (53)	0.56	52 (61)	0.68	46 (56)	0.35
Low	45 (52)		50 (56)		43 (56)	
Type of Treatment Received						
Cancer	47 (52)	0.91	51 (61)	0.94	46 (50)	0.78
BMT	47 (50)		52 (67)		46 (56)	
Treatment Phase						
Active	44 (43)	0.10	48 (56)	0.03	43 (39)	0.13
Surveillance	54 (58)		61 (69)		54 (61)	
Cancer Type						
Leukemia/lymph	51 (61)	0.3	56 (67)	0.2	51 (61)	0.18
Brain/solid	42 (37)		47 (50)		40 (33)	
Relapse status						
Yes	47 (59)	0.6	48 (61)	0.08	45 (56)	0.45
No	49 (52)		57 (67)		48 (50)	
Patient’s School						
Public school	49 (59)	0.17	54 (67)	0.07	41 (36)	0.26
Other	41 (40)		44 (44)		48 (61)	
Household Income						
High	42 (43)	0.04	49 (61)	0.57	40 (39)	0.04
Low	53 (63)		55 (67)		53 (61)	
COVID Vaccine status (self)						
0 or 1 dose	62 (65)	0.0002	67 (72)	0.0003	59 (61)	0.005
≥2 doses	41 (42)		45 (50)		41 (30)	
Influenza Vaccine status (self)						
≤2 doses in the last 5 years	51 (50)	0.14	55 (61)	0.15	51 (56)	0.09
3–5 doses in the last 5 years	43 (54)		48 (61)		42 (44)	

Numbers do not total 100% due to partial survey completion or rounding. Parental education level collapsed into high (college diploma, university degree, master’s, Ph.D. or professional degree) and low (high school diploma or below). Patient school collapsed into public and other (private, homeschool, not school age, other). Household income collapsed into high (>$50,000/year) and low (<$50,000/year).

**Table 4:** Vaccine hesitancy scores for parent vs. patient respondents.

Hesitancy Outcome	Parent, Mean (Median)	Patient, Mean (Median)	*p*-Value
VHS-Total	47 (52)	29 (17)	0.0036
VHS-COVID	51 (61)	24 (11)	0.0005
VHS-Flu	46 (56)	34 (31)	0.09

**Table 5:** Hesitancy outcomes for patient respondents based on demographics.

	VHS-Total		VHS-COVID		VHS-Influenza	
	Score, Mean (Median)	*p*-Value	Score, Mean (Median)	*p*-Value	Score, Mean (Median)	*p*-Value
Patient Gender						
Male	37 (31)	0.15	35 (31)	0.24	40 (36)	0.24
Female	20 (16)		17 (11)		27 (25)	
Parental Education						
High	22 (16)	0.8	20 (11)	0.65	28 (28)	0.62
Low	31 (17)		11 (11)		36 (33)	
Type of Treatment Received						
Cancer	20 (16)	0.005	16 (11)	0.016	25 (11)	0.003
BMT	64 (59)		67 (72)		74 (72)	
Treatment Phase						
Active	30 (20)	0.4	16 (8)	0.38	42 (33)	0.15
Surveillance	28 (16)		30 (11)		28 (28)	
Cancer Type						
Leukemia/lymph	17 (16)	0.83	13 (11)	0.74	20 (19)	0.49
Brain/solid	23 (16)		19 (11)		29 (28)	
Relapse status						
Yes	23 (16)	0.9	19 (6)	0.52	35 (33)	0.25
No	22 (16)		19 (11)		25 (25)	
School						
Public school	22 (16)	0.09	18 (11)	0.10	28 (28)	0.08
Other	51 (43)		54 (50)		56 (44)	
Household Income						
High	25 (16)	0.52	21 (11)	0.81	28 (28)	0.20
Low	30 (30)		28 (28)		39 (39)	
COVID Vaccine status (self)						
0 or 1 dose	66 (64)	0.02	67 (50)	0.02	67 (67)	0.03
≥2 doses	22 (16)		16 (11)		29 (28)	
Influenza Vaccine status (self)						
≤2 doses in the last 5 years	28 (17)	1.0	25 (11)	0.77	33 (33)	0.88
3–5 doses in the last 5 years	29 (18)		23 (11)		36 (28)	

Numbers do not total 100% due to partial survey completion or rounding. Parental education level collapsed into high (college diploma, university degree, master’s, Ph.D. or professional degree) and low (high school diploma or below). Patient school collapsed into public and other (private, homeschool, not school age, other). Household income collapsed into high (>$50,000/year) and low (<$50,000/year).

## Data Availability

Data for the study are available by contacting the corresponding author.
